# Non-pharmacological treatment of Alzheimer’s disease: an update

**DOI:** 10.3389/fnagi.2025.1527242

**Published:** 2025-02-13

**Authors:** Shaofen Wang, Haochen Xu, Guangdong Liu, Limei Chen

**Affiliations:** ^1^West China Xiamen Hospital of Sichuan University, Xiamen, Fujian, China; ^2^School of Medicine, University of Electronic Science and Technology of China, Chengdu, China

**Keywords:** non-pharmacological treatment, physical therapy, exercise therapy, cell therapy, nanoparticles, Alzheimer’s disease

## Abstract

Alzheimer’s disease (AD) is a neurodegenerative disorder that significantly impairs memory, cognitive function, and the ability to perform daily tasks. The pathological features of AD include β-amyloid plaques, neurofibrillary tangles, and neuronal loss. Current AD treatments target pathological changes but often fail to noticeably slow disease progression and can cause severe complications, limiting their effectiveness. In addition to therapies targeting the core pathology of AD, a more comprehensive approach may be needed for its treatment. In recent years, non-pharmacological treatments such as physical therapy, exercise therapy, cell therapy, and nanoparticles have shown great potential in mitigating disease progression and alleviating clinical symptoms. This article reviews recent advances in non-pharmacological treatment approaches for AD, highlighting their contributions to AD management and facilitating the exploration of novel therapeutic strategies.

## Graphical abstract

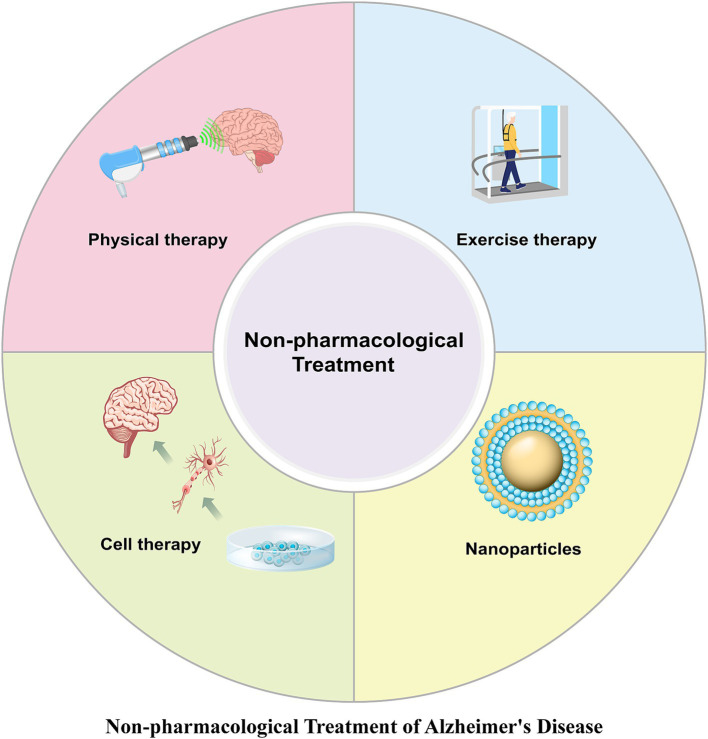

## 1 Introduction

Alzheimer’s disease (AD) is a chronic neurodegenerative disorder characterized by cognitive impairment, memory decline, and deficits in executive function (Beata et al., 2023). The main pathological features of AD include the deposition of β-amyloid (Aβ) protein, the accumulation of hyperphosphorylated tau protein, and neuronal death (Liu et al., 2023). In China, there are approximately 9.8 million patients with AD (Jia et al., 2020). With the increasing human lifespan, the number of AD patients is rapidly rising, leading to significant social and health challenges as well as a substantial economic burden (Livingston et al., 2020).

The drugs widely used in the clinical treatment of AD include acetylcholinesterase inhibitors and NMDA receptor antagonists, primarily for symptomatic treatment (Kabir et al., 2020). Currently, substantial progress has been made in the development of therapies targeting the core pathology of AD, focusing mainly on Aβ and tau proteins (Huimin et al., 2023; Yi et al., 2022). Aβ-related therapies include immunotherapy, BACE inhibitors, and γ-secretase inhibitors (Huimin et al., 2023). Lecanemab and donanemab, recently approved by the FDA, are anti-amyloid therapies that can slow cognitive decline but carry significant risks of brain swelling and bleeding (Couzin-Frankel, 2024). Tau-targeted therapies primarily consist of phosphorylation inhibitors, aggregation inhibitors, expression suppressors, and immunotherapies (Yi et al., 2022). However, these treatments can only delay disease progression, but they cannot reverse neuronal death or halt the progressive cognitive decline. Furthermore, the blood–brain barrier (BBB) significantly limits the bioavailability of these drugs, as it prevents effective doses from reaching the brain.

The pathogenic factors of AD are highly complex, and single treatments targeting AD pathology may be insufficient to slow disease progression. Combination therapy may represent a reasonable approach. This paper aims to summarize recent advances in non-pharmacological treatments for AD, including physical therapy, exercise therapy, cell therapy, and nanoparticles (NPs), and to discuss the research progress and challenges associated with these approaches. We review and analyze recent preclinical and clinical trials, focusing on new methods and technologies developed in recent years. These non-pharmacological treatments may serve as effective strategies for AD management, with minimal severe side effects. They may be applied directly to AD treatment or as adjuncts to pharmacological therapies, thereby potentially slowing or even reversing disease progression.

## 2 Non-pharmacological treatment

### 2.1 Physical therapy

Physical therapy primarily utilizes stimuli such as electricity, magnetic fields, sound, and light for treatment, offering the advantages of being non-invasive and highly safe (Shen et al., 2023). Physical therapy may represent a valuable therapeutic strategy for AD (Table 1).

**Table 1:** The methods, mechanisms, and effects of physical therapy.

Treatment methods		Patients/animal models	Treatment parameters	Mechanisms and therapeutic efficacy	Reference
Electrostimulation therapy	tDCS	Patients with early-stage AD	The current intensity was set at two mA, with each session lasting 30 min, once daily, for 6 months.	Improving cognitive function and regional brain glucose metabolism rate.	Im et al. (2019)
	tDCS	AD rat	The current intensity was 200 μA, with each session lasting 30 min, once daily, for 10 stimulation sessions.	Improving cognitive function and memory performance, with long-lasting effects lasting for up to 2 months.	Yang et al. (2019)
	TENS	Patients with AD	The internal frequency was 160 Hz, with a repetition rate of 2 Hz, pulse width of 100 μs, and duration per session of 30 min, once daily, for 6 weeks.	Improving non-verbal short-term memory.	Scherder and Bouma (1999)
	DBS	Patients with early-stage AD	The stimulation voltage was 3.0–3.5 V, with a frequency of 130 Hz and pulse width of 90 microseconds, sustained for 12 months.	DBS drives neural activity in the memory circuits and activates the brain’s default mode network, slowing the cognitive decline rate.	Laxton et al. (2010)
	DBS	Patients with AD	The stimulation voltage was 2.5 V, with a frequency of 130 Hz, pulse width of 210 microseconds, sustained for 12 months.	Memory scores remained stable compared to baseline, with increased metabolism in the medial temporal lobe.	Fontaine et al. (2013)
	DBS	Patients with mild to moderate AD	The stimulation voltage ranged from 2 to 4.5 V, with a frequency of 20 Hz, pulse width of 90–150 microseconds, sustained for 12 months.	Slight improvement or stabilization of specific AD-related symptoms.	Kuhn et al. (2015)
Magnetic stimulation therapy	rTMS	Patients with early-stage AD	The stimulation frequency was 20 Hz, with a stimulation duration of 2 s followed by a 28-s no-stimulation interval, lasting 20 min daily for 50 days.	Selective improvement in episodic memory, with no improvements observed in other cognitive domains.	Koch et al. (2018)
	rTMS	Patients with AD	The stimulation frequency was 20 Hz, with a stimulation duration of 2 s followed by a 28-s no-stimulation interval, lasting 20 min daily for 2 weeks.	Visual recognition memory and clock-drawing test scores showed significant improvement associated with elevated peripheral BDNF levels. Significant cognitive improvements were linked to enhanced network connectivity between the left parietal region and the hippocampus.	Velioglu et al. (2021)
	rTMS	Patients with AD	The stimulation frequency was 20 Hz, with a stimulation duration of 2 s followed by a 28-s no-stimulation interval, lasting 25 min daily for 20 days.	Improvement in language dysfunction.	Cotelli et al. (2011)
	rTMS-CT	Patients with AD	The stimulation frequency was 10 Hz, with a stimulation duration of 5 s followed by a 25-s no-stimulation interval, lasting 10 min daily for 20 days. All patients underwent CT for up to 1 h.	rTMS-CT may moderately prevent clinical and neuronal functional deterioration in the left DLPFC of AD patients.	Zhang et al. (2019)
	rTMS	5xFAD mice	The stimulation frequency was 20 Hz, with a magnetic stimulation intensity of 1.38 T, 100 pulses daily, with a 5-s interval between each pulse, sustained for 14 days.	Improvement in long-term memory for new objects and locations, enhancement of brain lymphatic system and meningeal lymphatic drainage efficiency, reduction of Aβ deposition, and decreased activation of microglia and astrocytes.	Lin et al. (2021)
Ultrasound therapy	Focused ultrasound	Patients with AD	Focused ultrasound was used to open the blood–brain barrier starting 2 h after each aducanumab infusion.	Focused ultrasound enhances the effects of aducanumab.	Rezai et al. (2024)
	Focused ultrasound	5xFAD mice	A 1 Hz burst repetition frequency, with a 10 ms burst duration, totaling 120 s, an average peak pressure of 0.25 MPa, administered over 6 weeks.	Improvement in cognitive dysfunction and working memory, with therapeutic effects lasting for 7 weeks. Additionally, focused ultrasound-mediated hippocampal BBB opening increased PKA phosphorylation.	Kong et al. (2023)
	Focused ultrasound	3xTg-AD mice	The pulse duration was 10 ms, with a repetition frequency of 5 Hz, PNP of 0.40 MPa, administered over 4 weeks.	Improvement in Aβ and tau pathology, as well as enhancement of spatial memory ability.	Karakatsani et al. (2023)
	Scanning ultrasound	APP23 mice	Parameters for the ultrasound delivery were 0.7-MPa peak rarefactional pressure, 10-Hz pulse repetition frequency, 10% duty cycle, 1 MHz center frequency, and 6-s sonication time per spot.	Reduction in Aβ plaque load and improvement in memory tasks.	Leinenga and Götz (2015)
	LIPUS	5xFAD mice	Center frequency = 1.875 MHz, pulse repetition frequency = 6.0 kHz, the number of cycles = 32 (17-us burst length), and spatial peak temporal average intensity = 99 mW/cm^2^.	Improvement in cognition related to cerebral blood flow and a reduction in microglia and Aβ plaques.	Eguchi et al. (2018)
	TPS	Patients with AD	The duration was approximately 3 μs, with an energy flux density of 0.2 mJ mm^−2^, a pulse repetition frequency of 5 Hz, 6,000 pulses per treatment session, and treatment lasting for 4 weeks.	Improve functional networks and cognitive abilities in AD, and reduce cortical atrophy in key brain regions associated with AD.	Popescu et al. (2021)
Phototherapy	PBM	TgF344-AD rats	The 808 nm continuous-wave low-level laser was administered for 2 min daily, three times a week, starting at 2 months of age and continuing until the mice reached 18 months.	Inhibition of neuroinflammation, improvement of mitochondrial dynamics, and suppression of oxidative damage. Enhancement of microglial recruitment around Aβ plaques, leading to improved Aβ clearance.	Yang et al. (2022)
	PBM	APP/PS1 mice	635 nm, 6 J/cm^2^, administered for 10 min daily over 30 days.	PBM inhibited Aβ-induced synaptic dysfunction and neuronal death, reducing amyloid burden, AMPA receptor endocytosis, dendritic damage, and inflammation, thereby rescuing memory deficits in mice. PBM activated ERK, which subsequently phosphorylated and stabilized MKP7, leading to the inactivation of JNK3.	Shen et al. (2021)
	PBM	APP/PS1 mice	Wavelength = 632.8 nm; Power = 92 mW; Irradiation time = 10 min; Beam area at the skin = 0.785 cm^2^; Number of treatments = 30; Treatment frequency = once per day.	Reduction in Aβ production and plaque formation improves memory and cognitive function.	Zhang et al. (2020)
	Gamma stimulation	5xFAD, APP/PS1 mice	40 Hz flicker (12.5 ms light on, 12.5 ms light off, 60 W) for 1 h	Increased co-localization of microglia with Aβ and reduced Aβ levels in the visual cortex.	Iaccarino et al. (2016)
	Gamma stimulation	5xFAD mice	40 Hz flicker (12.5 ms light on, 12.5 ms light off, 60 W) for 1.5 h	Promoted the influx of cerebrospinal fluid and efflux of interstitial fluid in the cortex of 5xFAD mice.	Murdock et al. (2024)	
	1,070 nm light	APP/PS1 mice	The pulse frequency was 10 Hz, with a wavelength of 1,070 ± 50 nm and an average power density of 25 mW/cm^2^. The irradiation lasted 6 min daily for a continuous period of 60 days.	Increased co-localization of microglia with Aβ, promoted angiogenesis and enhanced Aβ clearance.	Tao et al. (2021)
	Low-level laser irradiation	Aβ-infused SD rats.	808 nm continuous wave, administered for 2 min daily over a period of 4 weeks.	Inhibition of Aβ-induced hippocampal neurodegeneration and long-term spatial and recognition memory deficits. Restoration of mitochondrial dynamics and promotion of mitochondrial homeostasis; enhancement of antioxidant capacity while reducing oxidative damage; suppression of Aβ-induced reactive gliosis, inflammation, and tau hyperphosphorylation.	Lu et al. (2017)
Oxygen therapy	Oxygen	3xTg-AD mice	Oxygen concentration at 40%, administered for 20 min daily over a period of 2 months.	Alleviated protein synthesis damage and upregulated proteins associated with antioxidant defense.	Wang et al. (2017)
	HBOT	APP/PS1 mice	Exposure to 100% oxygen for 60 min daily in a hyperbaric chamber at 2.0 ATA, sustained for 28 days.	Significantly reduced Aβ accumulation and hippocampal neuroinflammation, increased hippocampal neurogenesis, and improved cognitive deficits.	Choi et al. (2019)
	HBOT	Patients with AD and MCI	Each treatment session included 20 min of pure oxygen inhalation (O₂ = 99.9%, oxygen pressure 0.4–0.7 MPa, oxygen flow rate 10–15 L/h), followed by a 15-min interval. Treatment was administered once daily for a duration of 20 days.	Significantly improved cognitive function in AD patients, as well as ameliorated brain glucose metabolism abnormalities.	Chen et al. (2020)
	HBOT	5xFAD mice	Administered 100% oxygen at 2 ATA for 60 min daily, 5 days a week, over a period of 4 weeks.	Increased the lumen diameter of small arteries and elevated cerebral blood flow, thereby helping to reduce hypoxia and decrease Aβ burden.	Shapira et al. (2021)

#### 2.1.1 Electrostimulation therapy

Transcranial direct current stimulation (tDCS) is a highly safe, non-invasive method for modulating cortical excitability. Both short-term and long-term tDCS have been shown to help delay disease progression in patients with AD (Gangemi et al., 2021). Additionally, tDCS can improve cognitive and language functions in AD patients and slow the deterioration of executive functions (Im et al., 2019). Repetitive anodal tDCS has been proven to enhance memory and cognitive functions, with its long-term effects lasting up to 2 months (Yang et al., 2019). A clinical study demonstrated that transcutaneous electrical nerve stimulation (TENS) can effectively improve emotional and memory function in AD patients (Scherder and Bouma, 1999). Deep brain stimulation (DBS) therapy can slow cognitive decline and improve glucose metabolism in the brain of AD patients (Laxton et al., 2010). One year of DBS treatment in AD patients can delay memory impairment (Fontaine et al., 2013). In one study, six AD patients underwent DBS treatment for 1 year, with four patients showing significant improvement in symptoms and no side effects (Kuhn et al., 2015). DBS treatment also slows the rate of hippocampal atrophy in AD patients (Sankar et al., 2015). tDCS, through the application of weak electrical currents flowing into neurons via the skull, can modulate neural plasticity, enhance learning and memory functions, and its effects can persist for a period after the stimulation ends (Laxton et al., 2010). It may also lead to morphological and phenotypic changes in astrocytes, thereby alleviating neuroinflammation (Yang et al., 2019). tDCS might affect the dynamic balance between ChAT and AChE, as well as influence the concentrations of GABA and glutamate neurotransmitters, potentially promoting more efficient information transmission (Stagg et al., 2009). Short-term side effects of tDCS, such as tingling, itching, headaches, and flashes, may occur but generally resolve quickly. However, if the stimulation exceeds the threshold, there is a risk of triggering seizures. While DBS can alleviate clinical symptoms in AD patients and slow down brain atrophy, its invasiveness limits patient acceptance, and the acceptance rate for DBS among patients is generally low. In contrast, tDCS is a promising non-invasive approach for treating memory impairment in early-stage AD patients. The safety and biological effects of electrical stimulation therapies require further and more comprehensive evaluation.

#### 2.1.2 Magnetic stimulation therapy

Repetitive transcranial magnetic stimulation (rTMS) is a non-invasive stimulation method that can modulate cortical activity and neuronal excitability, showing significant therapeutic potential (Zoicas et al., 2024). rTMS targeting the precuneus in AD patients can improve episodic memory and increase neuronal activity in the midbrain (Koch et al., 2018). Two weeks of 20 Hz rTMS over the left parietal lobe enhanced cognitive function and alleviated redox imbalance in AD patients (Velioglu et al., 2021). Another study demonstrated that 2 weeks of 20 Hz rTMS enhanced auditory comprehension in AD patients, with treatment effects lasting up to 8 weeks (Cotelli et al., 2011). Similarly, 10 Hz rTMS combined with cognitive training (CT) in AD patients improved cognitive and behavioral impairments, with findings suggesting that the left dorsolateral prefrontal cortex may be a more effective treatment target than the left lateral temporal cortex (Zhang et al., 2019). Combining rTMS with CT provides significant therapeutic benefits with high safety for patients with mild AD (Sabbagh et al., 2020). The combination of rTMS and CT is more effective in improving cognitive function than CT alone (Brem et al., 2020). In 5xFAD mice, rTMS has been shown to enhance lymphatic drainage in both the brain and meningeal lymphatic vessels, significantly reduce Aβ deposition, and inhibit the increase of microglia and astrocytes (Lin et al., 2021). rTMS may alter the polarization of neuronal cell membranes, thereby modulating neuronal network activity and synaptic plasticity. It may also influence underlying mechanisms such as the activation of glial cells, blood–brain barrier permeability, and vasodilation. These changes in mechanisms could lead to alterations in behavior and cognition (Zoicas et al., 2024). Although rTMS is generally well-tolerated, it is associated with a small risk of adverse effects, including seizures, mania, syncope, headaches, changes in hearing, neuropsychological alterations, and scalp electrode burns. The variability of rTMS effects and its therapeutic success rate are influenced by factors such as stimulation frequency and intensity, duration, coil shape and positioning, disease severity, and age. rTMS holds promise as an effective treatment for AD and, when combined with other interventions, may represent a novel approach to AD therapy.

#### 2.1.3 Ultrasound therapy

Ultrasound can open the BBB and modulate neural activity, with good tolerability and high safety, making it capable of alleviating AD pathology and improving cognitive and memory functions (Liu et al., 2021). Focused ultrasound can open the BBB and enhance the penetration of aducanumab (Rezai et al., 2024). In 5xFAD mice, focused ultrasound has been shown to restore memory function and synaptic plasticity over the long term (Kong et al., 2023). It also reduces Aβ and P-tau, improving spatial memory in 3xTg-AD mice (Karakatsani et al., 2023). Repeated scanning ultrasound therapy can reduce plaque burden in AD mice and enhance memory function (Leinenga and Götz, 2015). Low-intensity pulsed ultrasound (LIPUS) has been found to improve cognitive function while reducing Aβ plaques and microglial activation (Eguchi et al., 2018). LIPUS can inhibit neuroinflammation, reduce TNF-α and IL-1β, and alleviate memory deficits (Chen et al., 2019). Transcranial pulse stimulation (TPS) in AD patients has continuously improved neuropsychological scores without significant side effects (Beisteiner et al., 2020). TPS treatment can reduce cortical atrophy and significantly improve memory in AD patients (Popescu et al., 2021). The ability of ultrasound to open the BBB facilitates drug therapy, and ultrasound therapy may directly improve clinical symptoms and pathological changes in AD patients. Ultrasound therapy may induce skin reactions and pain as side effects. The parameters related to ultrasound need further optimization to establish standardized and safe protocols. In summary, ultrasound therapy is a non-invasive, highly safe therapeutic strategy for AD treatment.

#### 2.1.4 Phototherapy

Photobiomodulation (PBM) enhances mitochondrial function and reduces neuroinflammation, offering a non-invasive therapeutic approach that may hold great potential in the treatment of AD (Huang et al., 2024). PBM can reduce Aβ deposition, alleviate tau hyperphosphorylation, and mitigate neurodegeneration (Yang et al., 2022). Studies suggest that PBM can promote lymphatic system function, further aiding in the clearance of Aβ (Salehpour et al., 2022). PBM reduces Aβ deposition and inflammation, thereby mitigating neuronal death in AD mice (Shen et al., 2021). PBM can also activate the PKA/SIRT1 signaling pathway to reduce Aβ in AD mice, improving memory and cognitive abilities (Zhang et al., 2020). Gamma stimulation at 40 Hz in AD mice can reduce Aβ plaques and improve learning and memory function (Iaccarino et al., 2016). Additionally, 40 Hz gamma stimulation enhances the circulation of cerebrospinal fluid and interstitial fluid in 5xFAD mice, promoting neuronal activity and Aβ clearance (Murdock et al., 2024). In AD mice, 1,070 nm light at 10 Hz promotes microglial phagocytosis of Aβ, reducing Aβ deposition and improving learning and memory abilities (Tao et al., 2021). Low-level laser irradiation mitigates Aβ-induced mitochondrial damage, hippocampal neurodegeneration, and memory impairment (Lu et al., 2017). Near-infrared light therapy has been shown to improve cognitive function and activities of daily living in AD patients (Chen et al., 2023). The penetration ability of light through the skull and scalp into deep brain regions is limited. The inherent structural differences in the skulls of humans and experimental animals make it challenging to deliver sufficient light doses. Additionally, combining various light delivery methods may yield better outcomes (Salehpour et al., 2022). PBM may cause side effects such as skin burns, pain, redness, swelling, and allergic reactions. Phototherapy represents a non-pharmacological strategy that may help slow AD progression and improve clinical symptoms.

#### 2.1.5 Oxygen therapy

Hypoxia can induce neurodegeneration in AD patients, exacerbating Aβ, tau, and neuroinflammatory pathology (Liu et al., 2023). In APP/PS1 mice, blood oxygen saturation is significantly reduced even before the appearance of typical pathological changes (Wang et al., 2023a). Specifically, oxygen therapy enhances cognitive performance, reduces mitochondrial damage, alleviates protein synthesis impairment, and upregulates antioxidant defense-related proteins in AD mice (Wang et al., 2017). Furthermore, oxygen therapy reduces Aβ plaques in APP/PS1 mice, promotes hippocampal neurogenesis, and mitigates cognitive deficits (Choi et al., 2019). Hyperbaric oxygen therapy (HBOT) has shown effectiveness in improving cognitive function and correcting glucose metabolism dysregulation in AD patients (Chen et al., 2020). In 5xFAD mice, HBOT increases arteriolar lumen diameter and cerebral blood flow, thereby alleviating hypoxia, reducing Aβ plaques, and improving cognitive function (Shapira et al., 2021). In 3xTg mice, HBOT also reduces hypoxia, neuroinflammation, Aβ, and P-tau, thereby alleviating behavioral disorders (Shapira et al., 2018). HBOT improves learning and memory impairments in AD rats, reduces neuronal damage, and attenuates astrocyte activation (Zhao et al., 2017). HBOT can lead to oxygen toxicity, barotrauma, and decompression sickness. Furthermore, excessive oxygen levels may increase free radical production, exacerbate oxidative stress, and subsequently promote the pathology of AD (Liu et al., 2024). Oxygen therapy may regulate multiple pathological features of AD, including Aβ, P-tau, neuroinflammation, and cerebral blood flow, potentially serving as an effective therapeutic strategy for AD.

**Figure 1:**
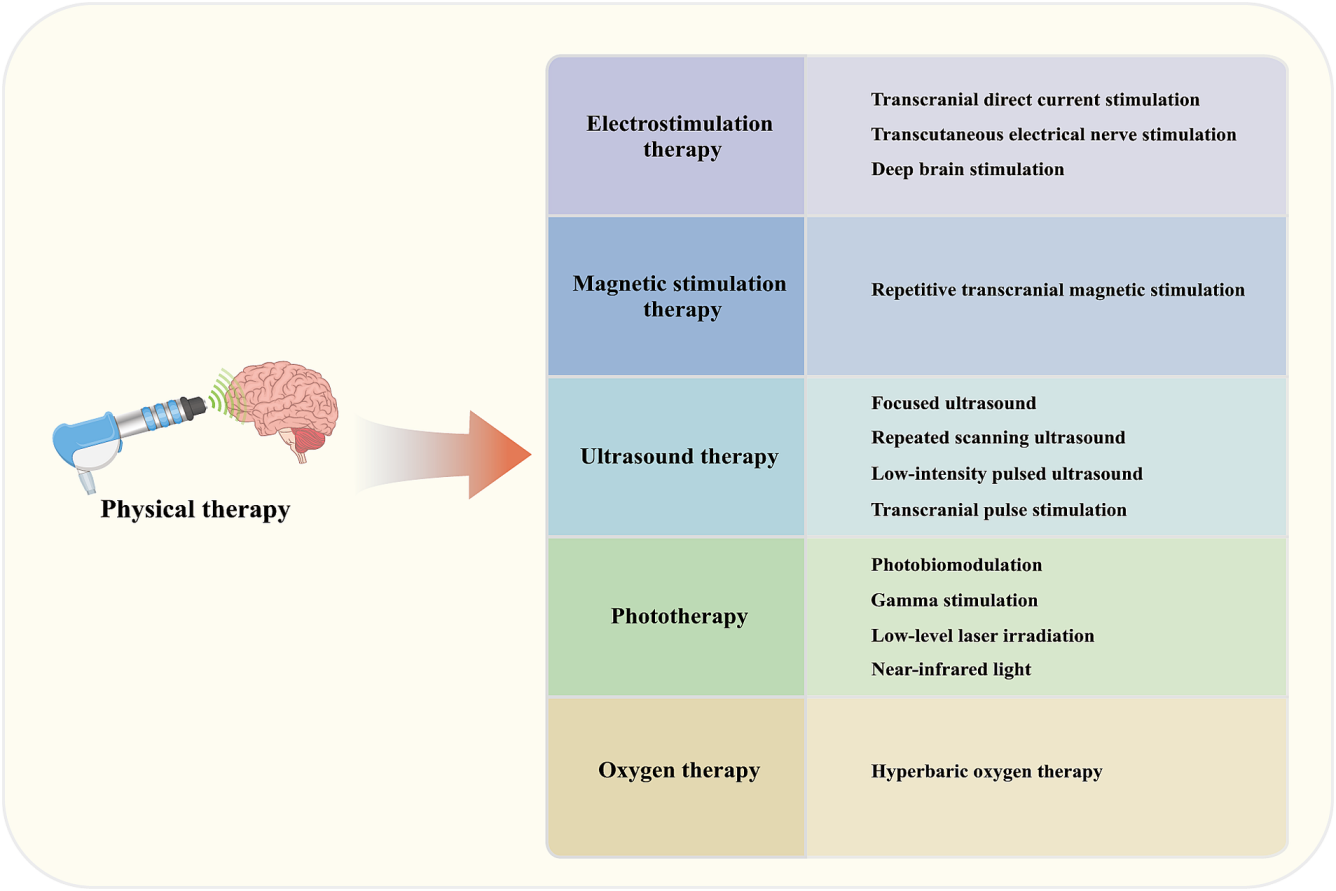
Physical therapy contributes to the treatment of AD.

Electrostimulation therapy, magnetic stimulation therapy, ultrasound therapy, Phototherapy, and oxygen therapy are all physical treatment modalities that can significantly alleviate the typical pathological changes of AD and improve clinical symptoms (Figure 1). Physical therapy holds great potential for the treatment of AD, and the discovery of new physical treatment methods may contribute to advancing therapeutic options for this condition.

Physical therapy primarily includes electrostimulation therapy, magnetic stimulation therapy, ultrasound therapy, Phototherapy, and oxygen therapy.

### 2.2 Exercise therapy

In addition to physical therapy, exercise therapy also plays a role in improving AD symptoms. Exercise can improve cognitive and executive functions, delaying the progression of clinical symptoms (De la Rosa et al., 2020; Wang et al., 2020). In patients with mild AD, exercise can alleviate depressive symptoms and enhance cognitive function (Hoffmann et al., 2016). The benefits of aerobic exercise on cognitive function in AD patients can be sustained over the long term (Ben Ayed et al., 2021). In patients carrying the APOE ε4 allele, exercise shows even more significant improvements in cognitive impairment (Jensen et al., 2019). Exercise training can delay spatial learning and memory deficits and reduce synaptic loss (Mu et al., 2022). Resistance exercise improves cognitive function, reduces Aβ and tau proteins, and alleviates neuroinflammation in APP/PS1 mice (Campos et al., 2023). Exercise increases levels of neurotrophic factors, reduces oxidative stress and neuroinflammation, and ameliorates AD pathology (López-Ortiz et al., 2021; Özbeyli et al., 2017). As a promising non-pharmacological treatment, exercise has the potential to delay the onset and progression of AD (Ryan and Kelly, 2016).

#### 2.2.1 Mitochondrial function

Exercise can mitigate the negative effects of AD, with mitochondria being an essential organelle in this process (Bernardo et al., 2016). Aerobic exercise effectively mitigates cognitive impairment in AD rats by reducing oxidative stress and mitochondrial dysfunction (Pantiya et al., 2023). Regular exercise reduces the expression of Aβ and P-tau proteins in AD mice, enhances brain energy metabolism, restores mitochondrial function, and improves spatial learning and exploratory abilities (Pang et al., 2019). Infusion of plasma from mice that exercised for 3 months into 3xTg-AD mice can improve neuronal plasticity and mitochondrial function, inhibit apoptosis, and ultimately enhance cognitive function (Kim et al., 2020). Exercise can reduce Aβ levels and enhance mitochondrial function, thereby improving learning and memory ability (Li et al., 2019). Regular swimming during pregnancy in female mice mitigates Aβ-induced damage by improving mitochondrial function (Klein et al., 2019). In 3xTg-AD mice, a combination of exercise and 40 Hz light flicker reduces Aβ and P-tau levels, and enhances neuronal plasticity and mitochondrial function, thereby improving learning and memory deficits (Park et al., 2020). Treadmill exercise improves mitochondrial function and reduces Aβ accumulation by enhancing PINK1/parkin-mediated mitophagy, thus improving learning and memory impairments (Zhao et al., 2023). Exercise may exert therapeutic effects by enhancing mitophagy and improving mitochondrial function (Zhao et al., 2021). Exercise therapy may counteract AD progression by restoring mitochondrial function.

#### 2.2.2 Neuroinflammation

Exercise can alleviate neuroinflammation, thereby improving neurodegeneration and cognitive impairment in AD patients (Valenzuela et al., 2020; Wang et al., 2023b). In AD mice, treadmill exercise significantly reduces the expression of Aβ and pro-inflammatory proteins (De Sousa et al., 2021). Resistance exercise also can decrease the levels of Aβ and various pro-inflammatory factors (Hashiguchi et al., 2020). In 3xTg-AD mice, exercise reduces hypothalamic neuroinflammation and improves glucose metabolism, which in turn alleviates neurodegeneration (Do et al., 2018). Exercise also decreases soluble Aβ, IL-1β, and TNFα levels in AD mice, exerting neuroprotective effects (Nichol et al., 2008). Swimming reduces Aβ and tau levels in AD rats while increasing levels of IL-10, BDNF, and NGF (Medhat et al., 2020). Early- and late-stage treadmill exercise diminishes microglial activation in AD mice (Ke et al., 2011). Treadmill exercise can reduce Aβ levels and astrocyte activation in AD mice (Zhang et al., 2018). Exercise may have beneficial effects on AD by reducing inflammation.

#### 2.2.3 Neurotrophic factors

Exercise plays a vital role in brain health and cognition by increasing levels of neurotrophic factors (Song, 2023). Aerobic exercise can improve memory function in AD patients by increasing serum BDNF levels (Erickson et al., 2011). Swimming has been shown to elevate BDNF and NGF levels in AD rats (Medhat et al., 2020). In AD rat models, exercise may improve cognitive abilities by raising neurotrophic factor levels and reducing oxidative stress (Belviranlı and Okudan, 2019). In 5xFAD mice, exercise reduces Aβ deposition, improves cognitive function, and increases levels of BDNF and synaptic markers (Choi et al., 2018). Exercise also regulates FNDC5/irisin expression, which is associated with elevated BDNF levels and reduced cognitive impairment (Hegazy et al., 2022). By enhancing the expression of neurotrophic factors, exercise exerts therapeutic effects on AD.

#### 2.2.4 Irisin

Irisin, a factor induced by exercise, is a cleaved form of FNDC5 released into the bloodstream after exercise and mediates the cognitive benefits of exercise in AD (Madhu et al., 2022). Cerebrospinal fluid levels of irisin are reduced in AD patients (Dicarlo et al., 2024). Exercise normalizes FNDC5/irisin expression, which is associated with reductions in Aβ and P-tau levels and improvements in cognitive impairment (Hegazy et al., 2022). Irisin is directly linked to Aβ pathology and cognition in AD patients, though its protective effects may be diminished by AD pathology (Kim et al., 2022; Lourenco et al., 2020). Irisin can improve cognitive deficits and ameliorate AD pathology in AD mice (Islam et al., 2021). FNDC5/irisin knockout mice experience memory decline, whereas overexpression of FNDC5/irisin improves memory damage (Zhou et al., 2019). FNDC5/irisin enhances synaptic plasticity and alleviates memory deficits in AD mice (Lourenco et al., 2019). Exercise-induced irisin increases the expression of Aβ-degrading enzymes by downregulating the ERK/STAT3 signaling pathway, significantly enhancing Aβ clearance in AD mice (Kim et al., 2023). Irisin generated through exercise improves cognitive function, reduces Aβ and P-tau, and exerts neuroprotective effects in AD.

**Figure 2:**
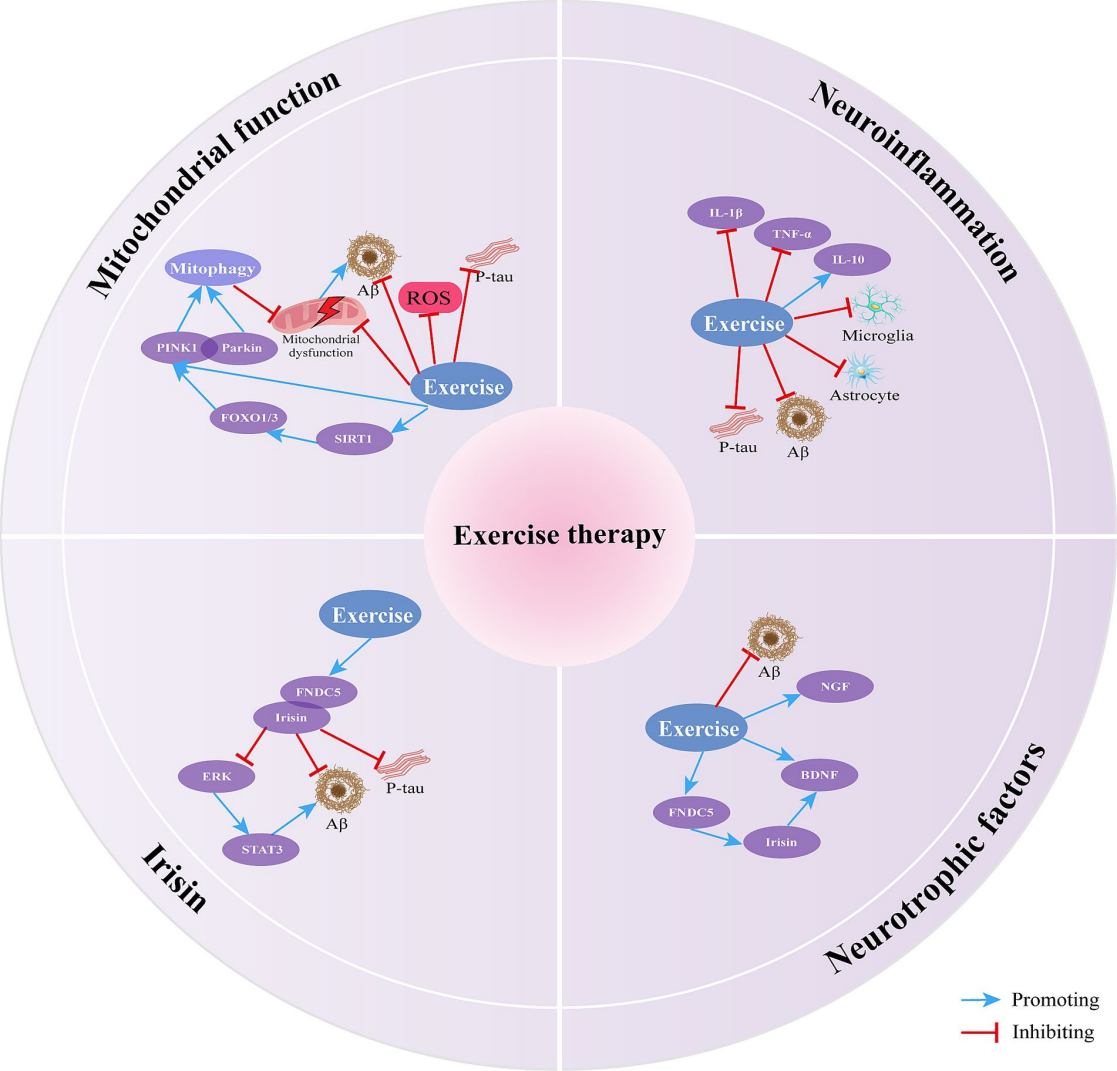
The mechanisms of exercise therapy in treating AD.

Exercise therapy can significantly improve the pathological and biochemical alterations in AD and alleviate clinical symptoms. It exerts therapeutic effects on AD by enhancing mitochondrial function, reducing neuroinflammation, increasing neurotrophic factor levels, and elevating irisin levels (Figure 2) (Table 2). Exercise therapy may pose risks such as falls and injuries. Age-related factors in AD patients also influence the application of exercise therapy. The persistence of exercise-induced effects, the optimal timing for initiating exercise, and the intensity of the intervention require further investigation (Ryan and Kelly, 2016).

**Table 2:** The methods, mechanisms, and effects of exercise therapy.

Treatment methods	Patients/animal models	Treatment parameters	Mechanisms and therapeutic efficacy	Reference
Supervised exercise	Patients with early-stage AD	Three times per week, 60 min per session, for 16 weeks.	Exercise can reduce neuropsychiatric symptoms in patients with mild AD and may also provide additional benefits for maintaining cognitive function.	Hoffmann et al. (2016)
Aerobic exercise	Patients with early-stage AD	Moderate-to-high intensity aerobic exercise was conducted for 1 h, 3 times per week, over 16 weeks.	APOE ε4 carriers derive greater benefits from exercise interventions.	Jensen et al. (2019)
Treadmill Exercise	3xTg-AD mice	The exercise protocol included 10 min at a speed of 12 m/min on a 0° incline, followed by 50 min at a speed of 15 m/min. Training sessions were conducted daily for 1 h, 5 days per week, for 12 weeks.	Improvement in spatial working memory, along with increases in synapse number, synaptic structural parameters, synaptophysin expression, axonal length, dendritic complexity, and dendritic spine density.	Mu et al. (2022)
Resistance training	APP/PS1 mice	The training was conducted 3 times per week, with each session consisting of 6–11 climbing trials, progressively overloaded over 4 weeks.	Improvement in behavior, corticosterone levels, and Aβ levels, with an increase in the number of microglia.	Campos et al. (2023)
High-intensity interval training	APP/PS1 mice	Mice ran at a speed of 8 m/min for 2 min, with the speed increasing by 1 m/min every 2 min until the mice could not maintain the treadmill pace for 10 consecutive seconds, over 12 weeks.	Improved exploratory behavior, spatial learning, and memory abilities. Reduced hippocampal Aβ burden, mitochondrial fragmentation, and improved hippocampal mitochondrial morphology.	Li et al. (2019)
Treadmill Exercise	APP/PS1 mice	Exercise protocol: 5 m/min for 5 min, 8 m/min for 5 min, 12 m/min for 30 min, and 5 m/min for 5 min, totaling 45 min per day, 5 days per week, for 12 weeks.	Enhanced PINK1/Parkin-mediated mitophagy improves mitochondrial function, reduces Aβ plaque accumulation, and improves learning and memory abilities.	Zhao et al. (2023)
Treadmill Exercise	APP/PS1 mice	During the first week, animals underwent 5 days of exercise therapy at a 10 m/min treadmill speed. In the second week, the daily exercise duration was increased by 10 min per day, gradually progressing from 20 min/day to 60 min/day. This was followed by 60 min/day of exercise maintained for three consecutive weeks.	Exercise improved spatial learning and memory, increased exploratory activity, and reduced anxiety.	Ke et al. (2011)
Swimming exercise	Rats that received intracerebroventricular injections of streptozotocin.	Rats swam for 15 min per day, with the duration increasing by 15 min daily until reaching 1 h of swimming time. Subsequently, they swam for 1 h daily, 5 days per week, for an additional 4 weeks.	The treatment normalized hippocampal FNDC5/irisin expression, which was associated with a reduction in Aβ and P-tau proteins, improved BDNF and insulin signaling, and alleviated cognitive impairments.	Hegazy et al. (2022)
Aerobic exercise	APP/PS1 mice	Swimming was then maintained at 60 min per day, 5 days per week, for 5 weeks.	FNDC5/irisin is a novel mediator of the beneficial effects of exercise on synaptic function and memory in AD models, playing a role in protecting/repairing synaptic function and preventing cognitive decline in AD.	Lourenco et al. (2019)

Exercise therapy affects AD by enhancing mitochondrial function, reducing neuroinflammation, increasing neurotrophic factor levels, and elevating irisin levels.

### 2.3 Cell therapy

In addition to physical therapy and exercise therapy, emerging cell-based therapies also represent a promising treatment approach for AD. Cell therapy, which promotes tissue regeneration by stimulating endogenous cells and replacing damaged cells, has emerged as a promising and effective treatment for AD (Khan et al., 2023).

#### 2.3.1 Stem cell transplantation

Stem cells possess the abilities of self-renewal, differentiation, and migration, enabling neuronal replacement and neurotrophic support, making them highly promising in the treatment of AD (Cao et al., 2024). In 5xFAD mice, induced pluripotent stem cells (iPSCs) can differentiate into glial cells, reducing cognitive impairment and Aβ deposition (Cha et al., 2017). Intranasal administration of iPSC-derived cortical neural stem cells (NSCs) secretomes alleviates memory deficits and Aβ plaque deposition in 5xFAD mice (Mo et al., 2023). Intranasal transplantation of human NSCs into APP/PS1 mice results in their differentiation into cholinergic neurons, reducing Aβ accumulation and ultimately alleviating cognitive dysfunction (Lu et al., 2021). Intravenous injection of mesenchymal stem cells (MSCs) can reduce microglial activation and pro-inflammatory factors in APP/PS1 mice (Naaldijk et al., 2017). Both NSC and MSC transplantation decrease hippocampal Aβ plaques and increase the number of microglial cells (Campos et al., 2022). Transplantation of stem cell-derived basal forebrain cholinergic neurons can induce functional recovery in AD animal models (Yue and Jing, 2015). In 3xTg-AD mice, human dental pulp stem cell transplantation exerts antioxidative stress and neuroprotective effects (Xiong et al., 2024). Transplantation of wild-type mouse hematopoietic stem and progenitor cells into 5xFAD mice reduces Aβ plaques, decreases neuroinflammation, and alleviates memory and cognitive impairments (Mishra et al., 2023). Stem cell transplantation can reduce Aβ deposition, P-tau, and neuroinflammation, secrete neurotrophic and growth factors, and reverse synaptic and neuronal damage, representing a promising therapeutic approach for AD (Chang et al., 2024).

#### 2.3.2 Extracellular vesicles

Extracellular vesicles (EVs), particularly exosomes, possess strong biocompatibility, easily cross the BBB, and have minimal side effects (Zhou et al., 2024). Exosomes in the brain regulate Aβ, tau, and neuroinflammation (Dinkins et al., 2017). Intranasal injection of EVs derived from cytokine-preconditioned MSCs into 3xTg mice suppresses microglial activation and increases dendritic spine density (Losurdo et al., 2020). Treatment of AD mice with MSC-derived exosomes improves glucose metabolism and cognitive function, reduces Aβ plaques, inhibits astrocyte activation, and increases the expression of memory- and synapse-related genes (Chen et al., 2021). Injecting exosomes from human umbilical cord MSCs into AD mice repairs cognitive dysfunction, aids in Aβ clearance, and regulates neuroinflammation (Ding et al., 2018). Exosomes from normoxic and hypoxia-preconditioned MSCs both improve cognitive and memory deficits in AD mice, reduce Aβ plaque deposition, modulate inflammation-related factors, and restore synaptic dysfunction (Cui et al., 2018). MSC-derived exosomes improve memory function in AD rats, reduce Aβ plaques and P-tau, promote neurogenesis, enhance synaptic function, and alleviate astrocyte proliferation (Ebrahim et al., 2024). Intraventricular injection of bone marrow MSC-derived exosomes suppresses excessive activation of hippocampal microglia and astrocytes, while reducing the expression of pro-inflammatory factor, Aβ, and P-tau (Liu et al., 2022). Injections of EVs from NSCs and MSCs enhance learning and memory functions in AD mice (Xia et al., 2022). Exosomes derived from NSCs significantly increase SIRT1 levels in AD mice, enhance mitochondrial biogenesis, and inhibit astrocyte activation (Li et al., 2024). After intravenous injection of iNSC-EVs, 5xFAD mice show improved cognitive function, reduced Aβ deposition, and diminished neuroinflammation (Gao et al., 2023). Plasma exosomes loaded with quercetin enhance drug bioavailability and brain targeting, inhibit the formation of P-tau, and alleviate cognitive dysfunction (Qi et al., 2020). EVs derived from young osteocytes significantly reduce Aβ plaques, mitigate synaptic and neuronal damage, and improve cognitive impairment (Jiang et al., 2022). Exosomes increase the solubility and bioavailability of curcumin, enhancing its penetration across the BBB. Exosomes derived from curcumin-pretreated cells reduce P-tau levels and neuronal death by activating the AKT/GSK3β pathway (Wang et al., 2019). EVs can reduce Aβ, P-tau, and neuroinflammation, while crossing the BBB, thereby playing a role in mitigating neuronal and synaptic damage (Rather et al., 2023).

### 2.4 NPs

In addition to cell therapy, NPs provide a promising delivery method for AD drugs. NPs are an important class of drug delivery materials characterized by their small size, which allows them to cross the BBB. They facilitate drug delivery across the BBB and enable sustained drug release, thereby improving the pharmacokinetics of therapeutic agents (Zhang et al., 2021).

#### 2.4.1 Polymeric NPs

Polymeric NPs encapsulate the drug core with a polymer shell, offering a flexible structure, nanoscale size, and good biodegradability. Poly (lactic-co-glycolic acid) (PLGA) has excellent biodegradability and biocompatibility, and is commonly used as a drug delivery carrier and tissue engineering scaffold. It is one of the most widely applied polymers approved by the FDA (Li and Jiang, 2018). Polyethylene glycol (PEG) is commonly utilized in nanoparticle formulations due to its ability to extend drug circulation time and slow clearance (Shi et al., 2022). PLGA NPs can reduce Aβ deposition and APP expression levels, decrease tau protein phosphorylation, and alleviate Aβ-induced neurotoxicity (Anand et al., 2022). Pioglitazone-loaded PLGA-PEG NPs cross the brain endothelium via endocytosis, improving memory deficits and reducing Aβ deposition in APP/PS1 mice (Silva-Abreu et al., 2018). PLGA-PEG-loaded fucoxanthin NPs enhance the bioavailability of fucoxanthin, reduce TNF-α and IL-1β levels, alleviate oxidative stress, and enhance the amelioration of cognitive impairment while reducing Aβ oligomer-induced neurotoxicity (Yang M. et al., 2021). Curcumin, known for its anti-Aβ, anti-inflammatory, and antioxidant properties, when loaded into PLGA-PEG NPs conjugated with B6 peptides, significantly improves spatial learning and memory abilities and reduces Aβ and P-tau formation (Fan et al., 2018). Curcumin-loaded chitosan and bovine serum albumin NPs effectively increase drug passage across the BBB, promote microglial activation, and accelerate Aβ peptide phagocytosis (Yang et al., 2018). Retro-inverso peptide inhibitor NPs efficiently inhibit Aβ aggregation and mitigate memory loss in AD mice (Gregori et al., 2017). Polymeric NPs are simple to produce and exhibit excellent biodegradability and biocompatibility. In AD, polymeric NPs can help inhibit core pathological features of the disease while providing antioxidant and anti-inflammatory effects.

#### 2.4.2 Metal NPs

Metal NPs easily cross the BBB and exhibit higher bioavailability, biocompatibility, and target specificity (Behera et al., 2023). Gold NPs (AuNPs) can inhibit the expression of inflammation factors induced by Aβ, reduce oxidative stress, and enhance cell viability (Chiang et al., 2021). AuNPs mitigate P-tau expression in AD mice, restore mitochondrial function and redox homeostasis, and prevent spatial memory impairment (Dos Santos Tramontin et al., 2020). D-glutathione stabilized AuNPs can cross the BBB in AD mice, inhibit Aβ aggregation, and improve behavioral deficits (Hou et al., 2020). Anthocyanin-loaded PEG-AuNPs enhance the neuroprotective effects of anthocyanins in AD mice, improving Aβ-induced memory impairment and synaptic dysfunction (Ali et al., 2017). Synthetic beta casein-coated iron oxide NPs can inhibit Aβ oligomerization and modulate neuroinflammation, apoptosis, and autophagy (Andrikopoulos et al., 2021). In APP/PS1 mice, superparamagnetic iron oxide NPs enhance the effect of curcumin in reducing Aβ and restoring memory deficits (Ruan et al., 2022). Zinc oxide NPs reduce Aβ formation, alleviate neuroinflammation, and improve memory and learning functions (Vilella et al., 2018). Cerium oxide NPs decrease Aβ and oxidative stress by regulating mitochondrial function (Dowding et al., 2014). Metal NPs may alleviate AD-related pathological changes and thus have potential therapeutic effects on AD.

However, metal NPs may also induce neurotoxicity and exacerbate AD pathology. Iron oxide NPs can cause oxidative stress and promote neuronal apoptosis in the brains of rats (Wu et al., 2013). Oleic acid-coated iron oxide NPs can disrupt cell membranes and damage the cell cycle, exerting cytotoxic effects (Fernández-Bertólez et al., 2018). Cobalt oxide NPs upregulate P-tau, NLRP3, and IL-1β expression, activating microglia and inducing neurotoxicity (Deng et al., 2021). Tin oxide NPs can induce Aβ protein formation and promote apoptosis through caspase-3 (Jaragh-Alhadad and Falahati, 2022). While metal NPs hold potential for AD treatment, their safety and efficacy require further investigation and evaluation.

#### 2.4.3 Liposome NPs

Liposomes are non-degradable and non-toxic, making them suitable as drug carriers to cross the BBB, enhance therapeutic efficacy, and reduce drug toxicity. Liposomes can serve as carriers for curcumin and neurotrophic factors, promoting drug delivery across the BBB, reducing Aβ plaque levels, and mitigating hippocampal neuronal damage (Kuo et al., 2017). Curcumin-loaded liposomes are non-toxic to SH-SY5Y cells and significantly reduce oxidative stress (Fernandes et al., 2021). PEG-curcumin liposomes can reduce Aβ formation in APP/PS1 mice (Mourtas et al., 2011; Mourtas et al., 2014). Transferrin-modified Osthole liposomes exhibit higher BBB penetration efficiency, improved bioavailability, prolonged circulation time, and significantly enhanced cognitive function. They reduce Aβ plaques and inhibit apoptosis in APP/PS1 mice (Kong et al., 2020). Transferrin-functionalized VB12-loaded liposomes delay Aβ fibril formation and disrupt mature fibrils (Andrade et al., 2022). Transferrin-Pep63-liposomes have BBB-targeting capabilities, significantly reducing Aβ load in APP/PS1 mice, enhancing microglial clearance, and improving cognitive deficits (Yang X. et al., 2021). Glutathione- and apolipoprotein E-grafted liposomes improve drug penetration through the BBB, facilitate the targeting of Aβ-damaged neurons, and reduce P-tau protein expression (Kuo et al., 2021). Intranasal administration of hydroxyl-α-sanshool liposomes enhances BBB crossing capacity, improving learning and memory in mice and alleviating hippocampal neuronal damage (Li et al., 2022). Metformin-loaded phosphatidylserine liposomes improve learning and memory in AD rats, reducing pro-inflammatory factor levels (Saffari et al., 2020). Liposomes can target pathological changes, delivering drugs to specific diseased areas, thus offering protective effects in AD.

We have summarized the relevant research on polymeric NPs, metal NPs, and liposomal NPs in AD. These NPs contribute to drug delivery and enhance therapeutic efficacy, demonstrating their significant potential in the treatment of AD. However, the safety and underlying mechanisms of these NPs still require further investigation.

## 3 Future direction

Non-pharmacological treatments are still exploratory, with certain limitations and areas requiring further investigation. Physical therapies, including electrostimulation, magnetic stimulation, ultrasound therapy, Phototherapy, and oxygen therapy, can delay pathological changes in AD and improve clinical symptoms. These therapies are non-invasive and have higher safety profiles. Yet, the optimal type of physical therapy, target regions, treatment duration, and frequency need further research. Additionally, the precise biological mechanisms through which physical therapy affects AD remain to be fully understood. Exercise therapy slows AD progression by regulating mechanisms such as mitochondrial function, neuroinflammation, and neurotrophic factors. However, further work is needed to develop suitable, sustainable exercise regimens, determine the most effective types of exercise, and identify the optimal frequency and duration for maximizing patient benefits. Cell therapy has made significant progress in AD animal models, with its regenerative potential positioning it as a promising cell replacement therapy for AD. Nonetheless, clinical trials are required to evaluate its efficacy and safety. The dosage and transplantation methods for stem cell therapy also need further study. Moreover, the mechanisms by which stem cells exert their effects in AD and strategies for effectively targeting delivery to specific tissues require deeper investigation. NPs offer several advantages, such as customizable surface properties, cross the BBB, targeted drug delivery, and enhanced absorption. However, the biodistribution and safety of NPs in AD remain areas of ongoing research. NPs can be combined with drug therapies to enhance therapeutic efficacy. Although significant progress has been made in NP-related studies in AD, long-term research is needed to transition from animal experiments to clinical trials.

Several challenges remain to be addressed. AD is a progressive disease, and long-term studies are required to verify the sustained efficacy of treatment approaches. Whether non-pharmacological therapies are effective in late-stage AD patients remains uncertain and warrants further investigation. The effectiveness, mechanisms, and safety of non-pharmacological treatments also require deeper research. In conclusion, non-pharmacological treatments hold great potential for AD therapy and could be used as adjunctive therapies to enhance the effectiveness of pharmacological treatments.

## 4 Conclusion

AD severely affects patients’ quality of life, with its incidence rising steadily each year. Although several typical pathological changes in AD have been identified, therapeutic approaches targeting these changes have not achieved ideal results or are associated with severe complications. This review discusses non-pharmacological treatments for AD, detailing advances in physical therapy, exercise therapy, cell therapy, and nanoparticle-based treatments. These non-pharmacological approaches may directly treat AD or serve as adjunctive therapies, offering additional benefits to AD patients.
